# Real-world patterns of inflammatory bowel disease treatment across adult and pediatric populations: a nationwide cohort study in South Korea

**DOI:** 10.3389/fmed.2026.1883050

**Published:** 2026-07-15

**Authors:** HyunJoo Lim, Ju Hwan Kim, Byong Duk Ye, Bin Hong, Bohyun Suh, Jae-Eun Lee, Yongjing Zhang, Hong Qiu, Ko Nakajo, Ju-Young Shin

**Affiliations:** 1 School of Pharmacy, Sungkyunkwan University, Suwon, Republic of Korea; 2 Department of Gastroenterology, University of Ulsan College of Medicine, Asan Medical Center, Seoul, Republic of Korea; 3 Inflammatory Bowel Disease Center, University of Ulsan College of Medicine, Asan Medical Center, Seoul, Republic of Korea; 4 Gerald Bronfman Department of Oncology, Faculty of Medicine and Health Sciences, McGill University, Montreal, QC, Canada; 5 Global Epidemiology, Johnson & Johnson, Seoul, Republic of Korea; 6 Global Epidemiology, Johnson & Johnson, Beijing, China; 7 Global Epidemiology, Johnson & Johnson, Raritan, NJ, United States; 8 Global Epidemiology, Johnson & Johnson, Tokyo, Japan; 9 Department of Clinical Research Design and Evaluation, Samsung Advanced Institute for Health Sciences and Technology, Sungkyunkwan University, Seoul, Republic of Korea

**Keywords:** advanced therapy, conventional therapy, inflammatory bowel disease, South Korea, treatment pattern

## Abstract

**Background/Aims:**

Although understanding real-world treatment patterns for inflammatory bowel disease (IBD) from initial diagnosis across age groups is essential, most studies focus on adult populations and advanced therapies.

**Methods:**

We conducted a descriptive study using a national healthcare database. Patients newly diagnosed with IBD between January 2014, and December 2022 were identified and classified into two groups by disease subtype: ulcerative colitis (UC) or Crohn's disease (CD). They were further stratified by age at the index date (pediatrics: <19 years, adults: ≥19 years). Then we examined patients' characteristics and overall treatment patterns.

**Results:**

A total of 60,181 individuals were identified as IBD patients with related medication use. Primarily, a younger age at diagnosis was observed among patients with CD, contributing to the predominance of CD within the pediatric population (proportion of CD patients: 71.6% in pediatrics and 24.8% in adults). Furthermore, pediatric-onset IBD was marked by a substantial burden of comorbidities (proportion of patients with ≥2 pediatric comorbidity index score: 41.7% in UC and 46.4% in CD) and a tendency toward earlier use of advanced therapies [median time to advanced treatment initiation, months (IQR): pediatrics vs. adults; 8.1 (2.7–18.9) vs. 16.9 (6.0–36.5) in UC; 4.2 (1.6–11.6) vs. 9.6 (3.0–27.3) in CD]. Secondarily, among restricted adult population with at least 1 year follow-up since first advanced agent prescription, cumulative persistence at 12 months was 76.9%−88.5% across the most recently approved agents (vedolizumab and ustekinumab).

**Conclusions:**

This study provides a comprehensive nationwide evaluation of patient characteristics and treatment patterns in adult and pediatric IBD patients in South Korea. These findings underscore the importance of tailoring treatment strategies according to disease subtype and age at diagnosis. Given that only tumor necrosis factor (TNF)-alpha inhibitors are currently approved for pediatric use and considering the emerging evidence regarding treatment persistence with newer agents, these findings may support consideration of expanding therapeutic options for pediatric patients.

## Introduction

Inflammatory bowel disease (IBD), encompassing ulcerative colitis (UC) and Crohn's disease (CD), is a chronic immune-mediated disorder characterized by alternating periods of remission and relapse (1, 2). These prolonged disease courses can lead to serious complications, including fistulas or cancer, thereby resulting in substantial morbidity and healthcare burden (3). The burden of IBD patients has increased worldwide, including in South Korea, where the prevalence approximately doubled between 2009 and 2019 (4). In addition, IBD frequently affects children and adolescents, who may experience growth impairment and other long-term complications due to disease progression (5). Given the rising prevalence of IBD and its potential lifelong consequences, optimized disease management is essential (6).

Over the past decades, treatment options for IBD have expanded from conventional to advanced therapies, including 5-aminosalicylates, steroids, immunomodulators, biologics, and small molecules, offering various alternative approaches for treatment-resistant patients. According to the national clinical guideline, initiating treatment with conventional therapy and escalating to advanced therapies is generally recommended (7). Although accumulating real-world evidence suggests that earlier use of advanced therapies may lead to improved clinical outcomes in pediatric patients (8–11), access to this treatment approach remains restricted under the current reimbursement system. Furthermore, approved advanced agents for pediatrics remain limited to tumor necrosis factor (TNF)-alpha inhibitors because the safety and efficacy of other newly developed agents have not been adequately established in this population. Therefore, gaining insights into real-world treatment patterns is key to supporting evidence-based decision-making.

Several studies have previously examined utilization patterns of IBD treatment in real-world settings (12–14). However, most have focused exclusively on adult populations and advanced therapies, rather than the overall treatment trajectory from initial diagnosis. Additionally, evidence regarding recently approved agents, such as integrin receptor antagonists, interleukin inhibitors, and Janus kinase (JAK) inhibitors, remains limited. To address these gaps, this study aimed to characterize the demographic and clinical features of IBD among both adult and pediatric patients and to assess overall treatment patterns.

## Methods

### IRB approval

This study was approved by Institutional Review Board of Sungkyunkwan University, South Korea (approval No. 2024-05-040). The need for informed consent was waived, as the study was conducted using anonymized claims data.

### Data source and study design

We conducted a retrospective cohort study using the National Health Insurance Service–National Health Information Database (NHIS-NHID). The NHIS is a mandatory administrative program for reimbursement purposes, and thus healthcare utilization records for all Korean residents are collected from birth until emigration or death (15). This longitudinal data includes demographic characteristics, socioeconomic status, inpatient and outpatient diagnoses, medication prescriptions, procedures, health screening records, and date of death. The diagnosis codes are followed by the International Classification of Diseases 10th Revision (ICD-10), with Rare and Intractable Disease (RID) program coding system. RID codes are specific identifiers to provide benefit coverage for patients with certain serious rare diseases. This study was reported following the Strengthening the Reporting of Observational Studies in Epidemiology (STROBE) guidelines (16).

### Study population

To identify patients newly diagnosed with IBD between January 1, 2014, and December 31, 2022, we included individuals with at least two diagnoses accompanied by RID code for IBD on the same day, applying a 1-year washout period. The index date was defined as the date of first diagnosis. We then classified them into two mutually exclusive cohorts based on the predominant subtype diagnoses within 1 year after the index date, considering the pathological characteristics and approved treatments differ substantially between UC and CD (hereafter referred to as the UC cohort and the CD cohort, respectively). If individuals could not be assigned to either group or did not receive IBD-specific medications (Supplementary Table S1) after the index date, they were excluded to ensure representativeness of the target diseases. This definition was based on a previously validated algorithm with high performance [sensitivity: 87.6%, positive predictive values (PPV): 98.5%] (17). We stratified patients by age at the index date based on the insurance coverage criteria in South Korea (pediatrics: <19 years; adults: ≥19 years). Using these cohorts, we analyzed their characteristics as well as overall treatment sequences.

Subsequently, to investigate the persistence rates and switching patterns of advanced therapies, we constructed the subcohort restricted to adult populations with at least 1 year of follow-up after the date of their first advanced therapy prescription, considering two factors: (1) the number of advanced therapies approved for pediatric patients in South Korea is limited to TNF-alpha inhibitors (infliximab for UC; infliximab or adalimumab for CD), and (2) the approval timing of these agents varies. Details of reimbursed medications for IBD, the reimbursement timeline of each agent for advanced therapy, and the reimbursement criteria are provided in Supplementary Tables S1, S2, Supplementary Figure S1, respectively.

### Patients' characteristics and overall treatment utilization sequences

Primarily, we observed the patients' characteristics and changes in overall treatment patterns over time. Comprehensive clinical and demographic characteristics were assessed at the time of initial diagnosis and at initiation of the first advanced therapy. We then followed patients from the date of their first diagnosis until the earliest occurrence of death, the end of the study period, or total colectomy (for UC only) to evaluate temporal changes in treatment trajectories.

### Persistence and switching of the first advanced treatment among adult populations

Secondarily, we assessed persistence and switching patterns of the first advanced therapy among the restricted adult population. Patients were followed from the date of their first prescription and were censored at death, the study end date, total colectomy (for UC only), 2 years after the start of follow-up, discontinuation, or switching, whichever occurred first. Non-persistence was defined as the absence of an additional prescription for the same agent within 90 days after the end date of the prior prescription. A 90-day grace period was selected based on the maximum reimbursable days' supply for advanced therapies in South Korea. The end date of each prescription was estimated by adding the recommended dosing interval to the prescription date, taking into account that days of supply are not available for injectable drugs (infliximab: 56 days; adalimumab: 14 days; golimumab: 28 days; ustekinumab: 84 days; vedolizumab: 56 days) (7, 18). For subcutaneous agents, the interval was multiplied by the number of units dispensed on the same day. If a different advanced agent was prescribed within 90 days after the end date of the initial agent, the case was defined as a switching event. Transitions between agents within the same class were also considered switching events, and only the first switching event per individual was included.

### Statistical analysis

Patients' characteristics were summarized as means [standard deviations (SD)] or medians [interquartile ranges (IQR)] for continuous variables, and as frequencies (percentages) for categorical variables. Treatment sequences from the time of first diagnosis were visualized at 6-month intervals over 3 years using Sankey diagrams. The frequencies and percentages of each treatment strategy were also presented in a table alongside the diagram. Finally, the risk of non-persistence and the number at risk were estimated at 3-month intervals using Kaplan–Meier analysis. All analyses were descriptive and were conducted without adjustment for clinical or demographic characteristics.

## Results

### Overall IBD population

**Figure 1:**
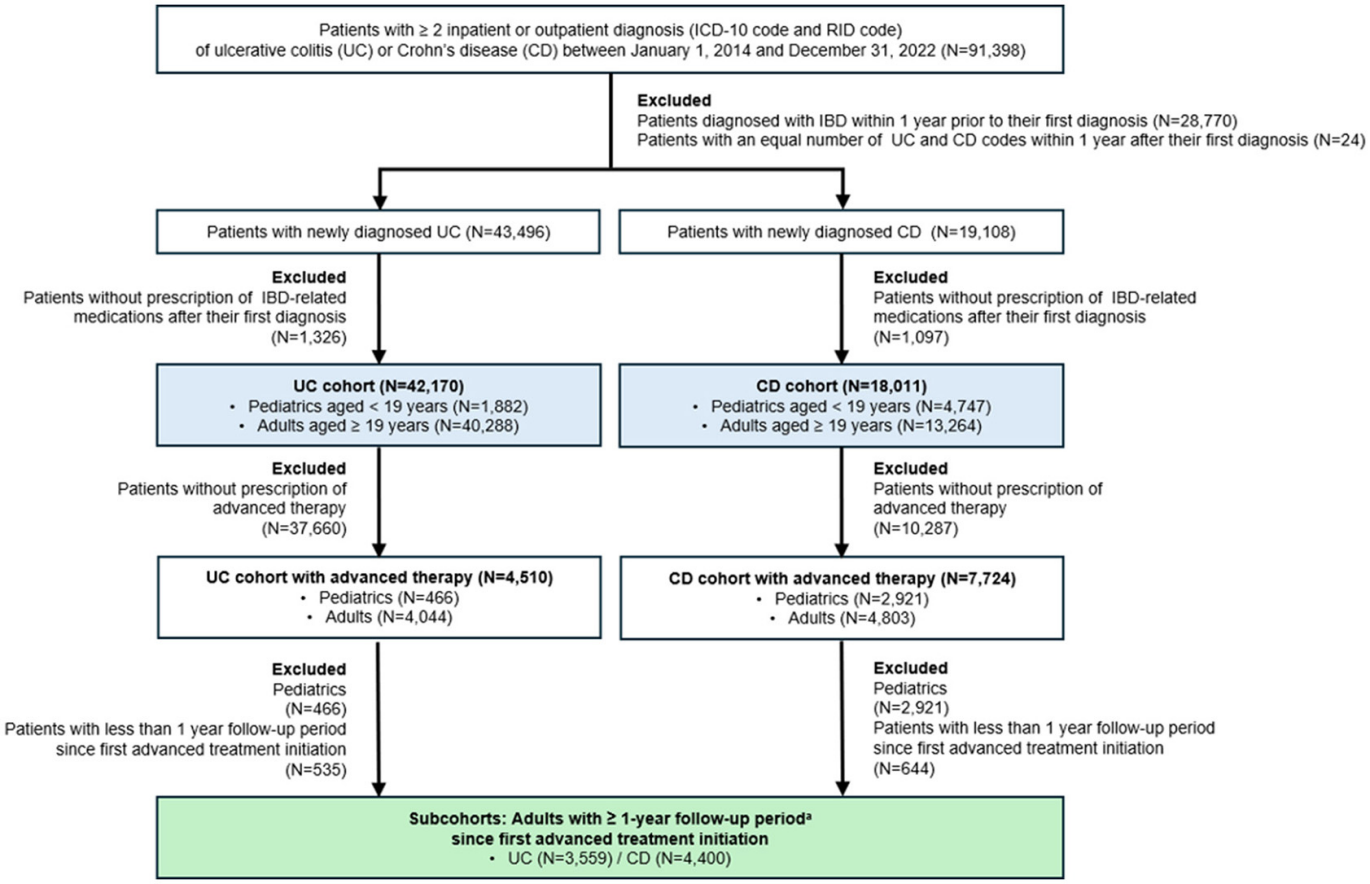
Study flow chart. ICD-10, International Classification of Diseases 10th revision; RID, rare and intractable disease; IBD; inflammatory bowel disease, UC; ulcerative colitis, CD; Crohn's disease. ^a^Patients were followed from their first diagnosis and censored at death, the study end date, or total colectomy (for UC only), whichever occurred first.

A total of 62,604 patients (UC, *N* = 43,496; CD, *N* = 19,108) were included in this study. After excluding those without any IBD-related medication prescriptions, 60,181 patients remained in the total cohort (hereafter, total cohort; UC, *N* = 42,170; CD, *N* = 18,011). When stratified by age group, 6,629 patients were pediatric (<19 years; UC, *N* = 1,882; CD, *N* = 4,747) and 53,552 were adults (≥19 years; UC, *N* = 40,288; CD, *N* = 13,264). Among adult patients, 7,959 (UC, *N* = 3,559; CD, *N* = 4,400) were followed for at least 1 year after initiation of advanced treatment (Figure 1).

### Patients' characteristics and treatment sequences by age group and IBD subtype

**Figure 2:**
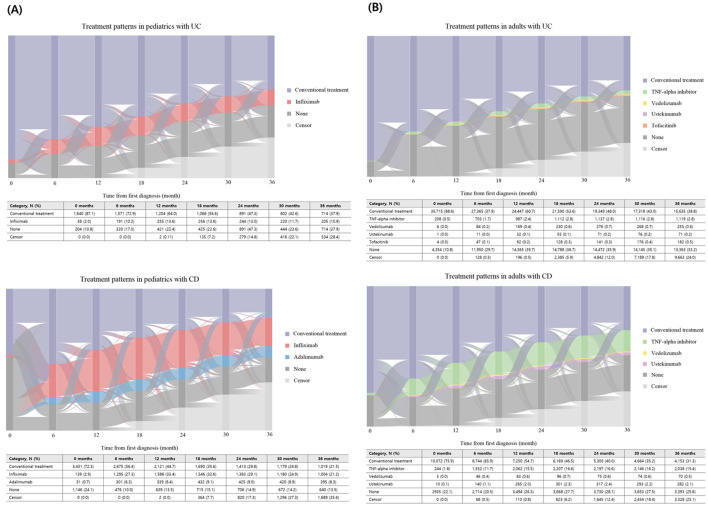
Overall treatment sequences over 36 months after diagnosis of UC or CD. **(A)** Pediatrics. **(B)** Adults. UC, ulcerative colitis; CD, Crohn's disease; TNF, tumor necrosis factor. Patients who received advanced therapy were grouped according to the type of advanced agent used, and those who received conventional treatment in combination were also included in these groups.

We observed multiple key patterns according to age at initial diagnosis and disease subtype. First, CD tended to occur at a younger age than UC. This was reflected by (1) a higher proportion of CD among pediatric patients [UC, *N* = 1,882 (28.4%); CD, *N* = 4,747 (71.6%)], and (2) a younger age at diagnosis among adults with CD than among those with UC (UC, 44.1 ± 15.5 years; CD, 35.0 ± 15.3 years). Second, pediatric patients exhibited a substantial comorbidity burden, with 41.7% and 46.4% of patients having a pediatric comorbidity index (PCI) score ≥2 in the UC and CD groups, respectively. Third, the use of advanced therapies was more common among pediatric patients, particularly those with CD (proportion of advanced treatment users: pediatrics vs. adults; UC, 24.8% vs. 10.0%; CD, 61.5% vs. 36.2%). Pediatric patients were more likely to initiate advanced therapies earlier than adult patients [median time to advanced therapy initiation: pediatrics vs. adults; UC, 8.1 months (IQR, 2.7–18.9) vs. 16.9 months (6.0–36.5); CD, 4.2 months (1.6–11.6) vs. 9.6 months (3.0–27.3) (Table 1)]. The proportion of patients initiating advanced therapy within 6 months was also higher among pediatric patients and in those with CD (pediatric vs. adult: UC, 10.2% vs. 2.0%; CD, 33.6% vs. 13.2%; Figure 2 and Supplementary Table S3). Lastly, TNF-alpha inhibitors, the earliest reimbursed class of advanced therapies, were the most frequently prescribed agents among the adult population. The distribution of advanced agents selected as first-line therapy largely reflected their reimbursement timelines (Table 1 and Supplementary Figure S1).

**Table 1:** Demographic and clinical characteristics of individuals with IBD.

Total number of patients	Pediatrics (*N* = 6,629)		Adults (*N* = 53,552)	
	UC (*N* = 1,882)	CD (*N* = 4,747)	UC (*N* = 40,288)	CD (*N* = 13,264)
Patients characteristics in total cohort (overall users)				
Sex, male (%)	1,136 (60.4)	3,514 (74.0)	24,570 (61.0)	9,732 (73.4)
Age (year) at first diagnosis				
Mean ± SD	15.4 ± 2.8	14.8 ± 2.8	44.1 ± 15.5	35.0 ± 15.3
Median (IQR)	16 (14–18)	15 (13–17)	43 (31–56)	30 (23–43)
19–39	NA	NA	17,196 (42.7)	9,284 (70.0)
40–59	NA	NA	15,715 (39.0)	2,660 (20.1)
≥60	NA	NA	7,377 (18.3)	1,320 (10.0)
Year at first diagnosis				
2014–2016	517 (27.5)	1,321 (27.8)	13,659 (33.9)	4,628 (34.9)
2017–2019	624 (33.2)	1,116 (23.5)	12,826 (31.8)	3,937 (29.7)
2020–2022	741 (39.4)	2,310 (48.7)	13,803 (34.3)	4,699 (35.4)
Region				
Seoul	344 (18.3)	812 (17.1)	9,326 (23.2)	2,794 (21.1)
Outside Seoul	1,538 (81.7)	3,935 (82.9)	30,962 (76.8)	10,470 (78.9)
Income level quartile				
Q1 (lowest)	317 (16.8)	811 (17.1)	7,650 (19.0)	2,771 (20.9)
Q2	257 (13.7)	689 (14.5)	8,550 (21.2)	3,031 (22.9)
Q3	401 (21.3)	1,007 (21.2)	10,343 (25.7)	3,182 (24.0)
Q4 (highest)	907 (48.2)	2,240 (47.2)	13,745 (34.1)	4,280 (32.3)
CCI score^a^				
Mean ± SD	NA	NA	1.3 ± 0.9	1.3 ± 0.9
Median (IQR)	NA	NA	1 (1–1)	1 (1–1)
0	NA	NA	17,627 (43.8)	5,275 (39.8)
1	NA	NA	19,336 (48.0)	6,780 (51.1)
≥2	NA	NA	3,325 (8.3)	1,209 (9.1)
PCI score^a^				
Mean ± SD	2.0 ± 1.5	2.2 ± 1.5	NA	NA
Median (IQR)	1 (1–3)	1 (1–3)	NA	NA
0	11 (0.6)	48 (1.0)	NA	NA
1	1,086 (57.7)	2,498 (52.6)	NA	NA
≥2	785 (41.7)	2,201 (46.4)	NA	NA
Follow-up period in years^b^				
Mean ± SD	5.1 ± 2.6	4.8 ± 2.8	5.4 ± 2.7	5.4 ± 2.7
Median (IQR)	5.0 (2.7–7.3)	4.1 (2.4–7.4)	5.4 (3.0–7.8)	5.4 (2.9–7.9)
Time to any treatment initiation in days				
Mean ± SD	5.5 ± 72.7	8.2 ± 54.2	12.1 ± 113.2	20.1 ± 144.9
Median (IQR)	0 (0–0)	0 (0–0)	0 (0–0)	0 (0–0)
No. of patients initiating advanced treatment	466 (24.8)	2,921 (61.5)	4,044 (10.0)	4,803 (36.2)
Patients characteristics in sub cohort (among advanced treatment users)				
Sex, male (%)	273 (58.6)	2,125 (72.8)	2,702 (66.8)	3,531 (73.5)
Age (year) at first advanced treatment				
Mean ± SD	15.9 ± 3.2	15.4 ± 3.2	43.7 ± 15.7	32.9 ± 13.0
Median (IQR)	16 (14–18)	16 (13–18)	43 (30–56)	29 (23–38)
19–39	NA	NA	1,789 (44.2)	3,701 (77.1)
40–59	NA	NA	1,522 (37.6)	812 (16.9)
≥60	NA	NA	733 (18.1)	290 (6.0)
Region				
Seoul	74 (15.9)	500 (17.1)	873 (21.6)	947 (19.7)
Outside Seoul	392 (84.1)	2,421 (82.9)	3,171 (78.4)	3,856 (80.3)
Income level quartile				
Q1 (lowest)	80 (17.2)	543 (18.6)	794 (19.6)	1,112 (23.2)
Q2	68 (14.6)	404 (13.8)	891 (22.0)	1,154 (24.0)
Q3	98 (21.0)	587 (20.1)	1,061 (26.2)	1,117 (23.3)
Q4 (highest)	220 (47.2)	1,387 (47.5)	1,298 (32.1)	1,420 (29.6)
CCI score^a^				
Mean ± SD	NA	NA	1.3 ± 1.0	1.3 ± 0.8
Median (IQR)	NA	NA	1 (1–1)	1 (1–1)
0	NA	NA	1,244 (30.8)	1,795 (37.4)
1	NA	NA	2,388 (59.1)	2,640 (55.0)
≥2	NA	NA	412 (10.2)	368 (7.7)
PCI score^a^				
Mean ± SD	2.6 ± 1.5	2.5 ± 1.5	NA	NA
Median (IQR)	3 (1–3)	3 (1–3)	NA	NA
0	0 (0.0)	7 (0.2)	NA	NA
1	147 (31.6)	1,117 (38.2)	NA	NA
≥2	319 (68.5)	1,797 (61.5)	NA	NA
Time to advanced treatment initiation in months				
Mean ± SD	14.6 ± 18.8	11.0 ± 17.0	24.9 ± 24.7	19.6 ± 23.6
Median (IQR)	8.1 (2.7–18.9)	4.2 (1.6–11.6)	16.9 (6.0–36.5)	9.6 (3.0–27.3)
Type of first advanced treatment				
Infliximab	466 (100.0)	2,255 (77.2)	1,710 (42.3)	2,684 (55.9)
Adalimumab	NA	666 (22.8)	803 (19.8)	1,181 (24.6)
Golimumab	NA	NA	270 (6.7)	NA
Vedolizumab	NA	NA	848 (21.0)	235 (4.9)
Ustekinumab	NA	NA	160 (4.0)	703 (14.6)
Tofacitinib	NA	NA	253 (6.3)	NA
Preceding conventional treatment				
5-ASA	445 (95.5)	2,414 (82.6)	3,948 (97.6)	4,189 (87.2)
Corticosteroids	445 (95.5)	2,346 (80.3)	3,878 (95.9)	4,174 (86.9)
Immunomodulators	379 (81.3)	2,592 (88.7)	2,900 (71.7)	4,302 (89.6)

IBD, inflammatory bowel disease; UC, ulcerative colitis; CD, Crohn's disease; SD, standard deviation; IQR, interquartile range; CCI, Charlson comorbidity index; PCI, pediatric comorbidity index; 5-ASA, 5-aminosalicylic acid.

^a^To account for differences between patient populations, we used the CCI in adults and the PCI in children to evaluate comorbidity burden.

^b^Patients were followed from their first diagnosis date until the earliest of the following: death, the date of total colectomy receipt (UC only), or end of the study period.

### Persistence and switching patterns of first advanced treatment among adult populations

**Figure 3:**
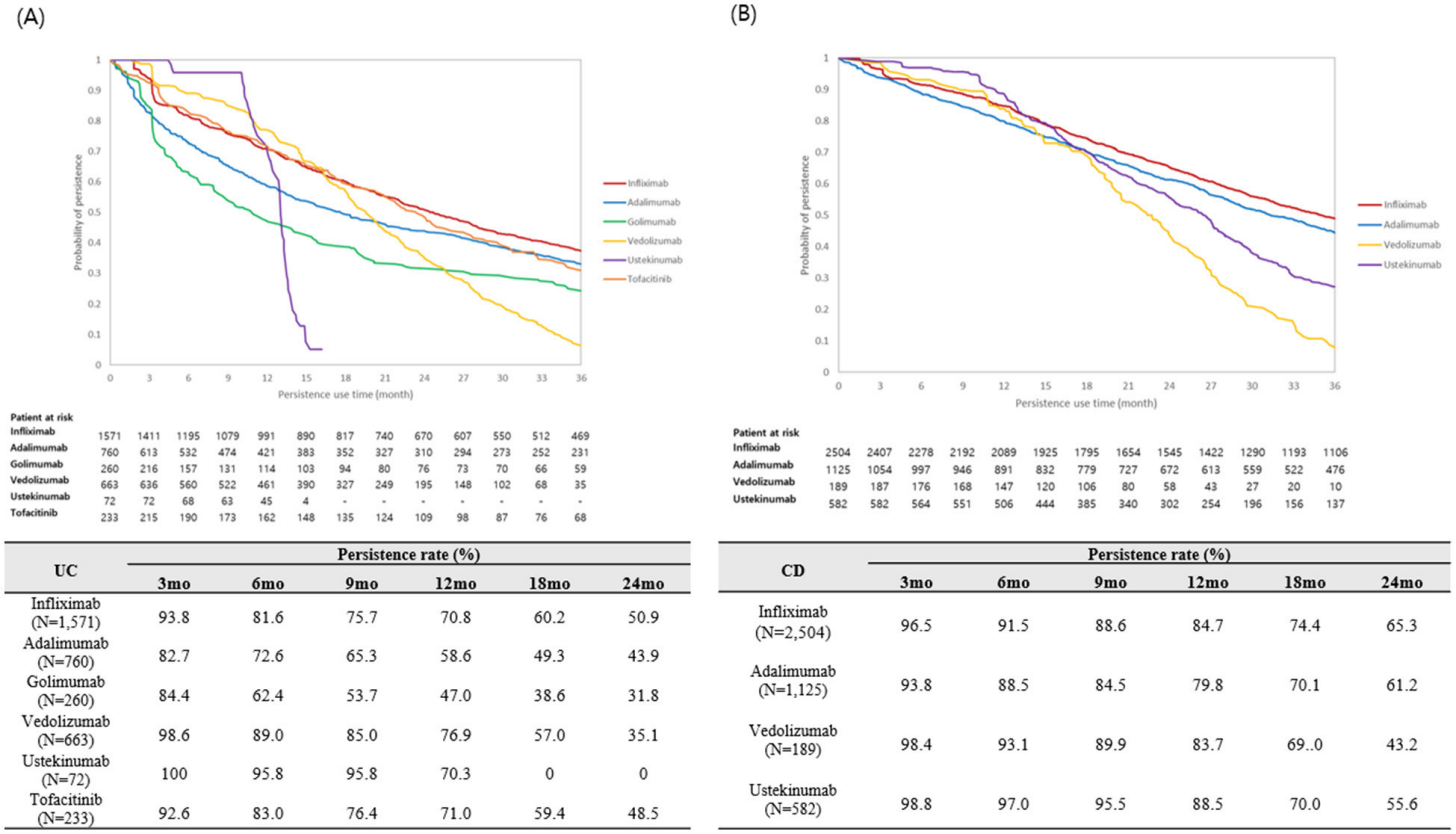
Kaplan–Meier curve for persistence of first advanced treatment among adult populations. **(A)** UC. **(B)** CD. UC, ulcerative colitis; CD, Crohn's disease.

Figure 3 presents the persistence rates of the first advanced therapy, stratified by agent, over the 2-year follow-up period. Among UC patients, the cumulative probability of persistence at 12 months was highest for vedolizumab (76.9%), and ustekinumab also showed favorable persistence compared with other agents. A similar pattern was also observed in CD patients (ustekinumab, 88.5%; vedolizumab, 83.7%). However, vedolizumab demonstrated a higher switching rate than other agents in the CD group (Infliximab, 2.6%; vedolizumab, 21.7%; ustekinumab, 4.9%), whereas comparable switching rates were observed across intravenous agents in the UC group (Infliximab, 11.5%; vedolizumab, 13.3%; ustekinumab, 12.5%; Table 2).

**Table 2:** Switching patterns between advanced therapy among adult population.

	First treatment						
	Infliximab	Adalimumab	Golimumab	Vedolizumab	Ustekinumab	Tofacitinib	Total
	(*n* = 1,571)	(*n* = 760)	(*n* = 260)	(*n* = 663)	(*n* = 72)	(*n* = 233)	(*n* = 3,559)
(A) Ulcerative colitis (UC)							
Total switching cases, n (%)^a^	**180 (11.5)**	**41 (5.4)**	**17 (6.5)**	**88 (13.3)**	**9 (12.5)**	**10 (4.3)**	**345 (9.7)**
Time to switching, median (IQR), mo	**4.6 (2.4–9.0)**	**3.7 (1.8–7.7)**	**3.5 (2.6–6.9)**	**8.2 (3.1–12.9)**	**6.8 (6.4–7.9)**	**5.0 (3.0–8.2)**	
Secondary treatment, n (%)							
Infliximab	NA	23 (56.1)	11 (64.7)	48 (54.5)	2 (22.2)	5 (50.0)	89 (25.8)
Adalimumab	69 (38.3)	NA	1 (5.9)	7 (8.0)	1 (11.1)	1 (10.0)	79 (22.9)
Golimumab	11 (6.1)	2 (4.9)	NA	0 (0.0)	0 (0.0)	1 (10.0)	14 (4.1)
Vedolizumab	26 (14.4)	4 (9.8)	3 (17.7)	NA	4 (44.4)	3 (30.0)	40 (11.6)
Ustekinumab	16 (8.9)	2 (4.9)	1 (5.9)	19 (21.6)	NA	0 (0.0)	38 (11.0)
Tofacitinib	58 (32.2)	10 (24.4)	1 (5.9)	14 (15.9)	2 (22.2)	NA	85 (24.6)
**First treatment**							
		Infliximab	Adalimumab	Vedolizumab	Ustekinumab	Total	
		**(*****n*** = **2,504)**	**(*****n*** = **1,125)**	**(*****n*** = **189)**	**(*****n*** = **582)**	**(*****n*** = **4,400)**	
(B) Crohn's disease (CD)							
Total switching cases, n (%)^a^		**66 (2.6)**	**16 (1.5)**	**41 (21.7)**	**29 (4.9)**	**152 (3.5)**	
Time to switching, median (IQR), mo		**10.2 (6.3–16.6)**	**9.5 (6.6–17.3)**	**13.3 (11.3–18.4)**	**13.7 (9.4–16.2)**		
Secondary treatment, n (%)							
Infliximab		NA	8 (50.0)	23 (56.1)	23 (79.3)	54 (35.5)	
Adalimumab		41 (62.1)	NA	5 (12.2)	3 (10.3)	49 (32.2)	
Vedolizumab		3 (4.5)	0 (0.0)	NA	3 (10.3)	6 (3.9)	
Ustekinumab		22 (33.3)	8 (50.0)	13 (31.7)	NA	43 (28.3)	

IQR, interquartile range; mo, months.

^a^We assumed that a patient had switched to another advanced treatment agent if a different agent was dispensed within 90 days after end date of their first one.

## Discussion

In this nationwide population-based cohort study, we examined the patient characteristics and treatment patterns of IBD, spanning all age groups and the full range of approved medications. Several notable features emerged according to IBD subtype, age at initial diagnosis, and the type of advanced therapy. Patients with CD tended to be diagnosed at a younger age, resulting in a higher proportion of CD among pediatric patients. Pediatric-onset IBD was also characterized by a substantial comorbidity burden and more frequent early initiation of advanced therapies in the disease course. In addition, among adult patients receiving advanced therapies, ustekinumab and vedolizumab, the most recently approved agents, showed higher 12-month persistence rates than other therapies.

Our findings are in line with previous research supporting the need for careful clinical management of younger patients with IBD. Approximately one-quarter of IBD cases present before the age of 20, with incidence rates declining sharply thereafter, and CD accounts for a higher proportion of cases among pediatric patients (4, 5). These patients tend to experience a more aggressive disease course, which has led to continued emphasis on early initiation of advanced therapies (19–21). A recent U.S. claims-based study also reported that more than one-third of newly diagnosed pediatric IBD patients received biologic monotherapy within the first year, with a shorter interval between diagnosis and treatment initiation compared with adults (36 days vs. 62 days, respectively) (22). However, despite the increasing disease burden of IBD in South Korea, evidence on age-specific disease characteristics and treatment patterns remains limited, particularly among pediatric patients. Therefore, the present study expands the existing evidence base by providing quantitative data on age-specific clinical characteristics and treatment trajectories following diagnosis among patients with IBD in South Korea. Subsequently, we observed high persistence rates among the newly approved advanced therapies. This trend is consistent with the previous findings from various countries (13, 23, 24). In a Korean cohort study, first-line ustekinumab therapy in CD was associated with a lower risk of non-persistence than infliximab [adjusted hazard ratio (aHR), 0.69; 95% Confidence interval (95% CI), 0.49 to 1.00] (13). Likewise, a cohort study using the Japan Medical Data Center database also reported the highest 6-month persistence for ustekinumab after IBD diagnosis (UC: 95.1% for ustekinuamb vs. 68.0% for adalimumab, 76.0% for infliximab, 73.9% for golimumab, 82.0% for vedolizumab, 68.1% for tofacitinib; CD: 94.7% for ustekinumab vs. 91.7% for adalimumab, 94.1% for infliximab, 78.1% for vedolizumab) (24). Our findings further support and extend this growing body of evidence by demonstrating similar persistence patterns in a nationwide South Korean population using recent data.

However, our findings merit closer attention to the persistence of advanced therapy, and several issues remain to be addressed in future studies. First, although vedolizumab showed high persistence rates in both UC and CD patients, it also presented a relatively high switching rate. This apparent inconsistency has also been reported in previous studies. While the Japanese study reported that vedolizumab had the lowest 6-month persistence among advanced treatments in patients with CD, (24) studies in the US and South Korea demonstrated persistence rates comparable to those of other biologics (13, 25). In addition, the sample sizes and follow-up periods for recently approved agents were relatively smaller and shorter, respectively, than those for other therapies. Therefore, further investigation using longer-term longitudinal data is warranted to better characterize difference across agents. Second, data in pediatric populations remain scarce. Several electronic health record-based observational studies have shown that ustekinumab demonstrated favorable efficacy and safety profiles in children, suggesting the potential to expand treatment options (26, 27). Vedolizumab has also been reported to have a low likelihood of loss of response in pediatric patients (11). However, because treatment options for children and adolescents remain more restricted than those for adults, the available evidence is largely derived from small off-label cohorts, which limits generalizability. Meanwhile, multiple studies have demonstrated the benefits of early biologic intervention in pediatric IBD, including reduced corticosteroid requirements, more rapid symptom improvement, lower C-reactive protein levels, and improved endoscopic mucosal healing (9, 10, 21, 28). Therefore, additional evidence is needed to support broader therapeutic options for younger patients.

### Limitations

This study has some limitations. First, some agents were only recently approved as first-line biologics (Supplementary Figure S1), so the sample size was relatively small and the follow-up period was shorter than for TNF-alpha inhibitors. This may have limited the comparability of findings across agents. Second, several features inherent to the data source warrant caution in the interpretation of our findings: (1) prescriptions for non-reimbursed medications could not be identified. Therefore, the possibility that patients received other advanced therapies not approved for IBD during the study period cannot be completely ruled out. (2) The evaluation of treatment patterns among pediatric patients was constrained because newer advanced therapies were not approved for this population during the study period. (3) Important clinical indicators, such as endoscopic disease activity, C-reactive protein levels, and the Harvey–Bradshaw Index, and the reasons for treatment non-persistence or switching were not captured. As these factors may influence treatment selection and clinical decision-making, the interpretation of findings related to treatment persistence and the timing of advanced therapy initiation should be made with caution. Third, we applied a fixed 90-day grace period to assess treatment persistence and switching patterns. However, this approach may have introduced exposure misclassification because induction and maintenance dosing intervals vary across medications. Fourth, all analyses were descriptive in nature and were conducted without adjustment for factors such as disease severity, comorbidity burden, and physician practice patterns, which may have influenced treatment patterns and outcomes. Therefore, the findings should be interpreted with caution and should not be considered evidence of causal relationships or differential treatment preferences.

## Conclusions

This study provides the first nationwide evaluation of patient characteristics and treatment patterns among adult and pediatric patients with IBD in South Korea. CD was more commonly diagnosed at a younger age than UC, and pediatric patients tended to initiate advanced therapies earlier in the disease course. These findings highlight the importance of considering both disease subtype and age at diagnosis when developing treatment strategies. Given the limited therapeutic options currently approved for pediatric patients and the growing body of evidence regarding newer agents, further research is warranted to better inform treatment decision-making and future therapeutic approaches in this population.

## Data Availability

The data analyzed in this study is subject to the following licenses/restrictions: Data generated and/or analyzed during the current study cannot be shared publicly due to the data-sharing policy of the National Health Insurance Service (NHIS) of Korea. Requests to access these datasets should be directed to https://nhiss.nhis.or.kr.
