# Supplementary material for: Real-world patterns of inflammatory bowel disease treatment across adult and pediatric populations: a nationwide cohort study in South Korea
Source: Front Med (Lausanne). 2026 Jul 15;13:1883050. doi: 10.3389/fmed.2026.1883050 (PMC13414261; doi:10.3389/fmed.2026.1883050)
Supplement: Supplementary file 1 [file Data_Sheet_1.docx]

**Supplementary Materials**

**Supplementary Figure S1.** National Health Insurance Service (NHIS) reimbursement status for advanced treatment

**Supplementary Figure S2.** study design diagram

**Supplementary Table S1.** List of reimbursed IBD-specific medications

**Supplementary Table S2.** Reimbursement criteria for advanced therapy

**Supplementary Table S3.** Temporal changes in IBD therapy since the first diagnosis

**Supplementary Figure S1.** National Health Insurance Service (NHIS) reimbursement status for advanced treatment


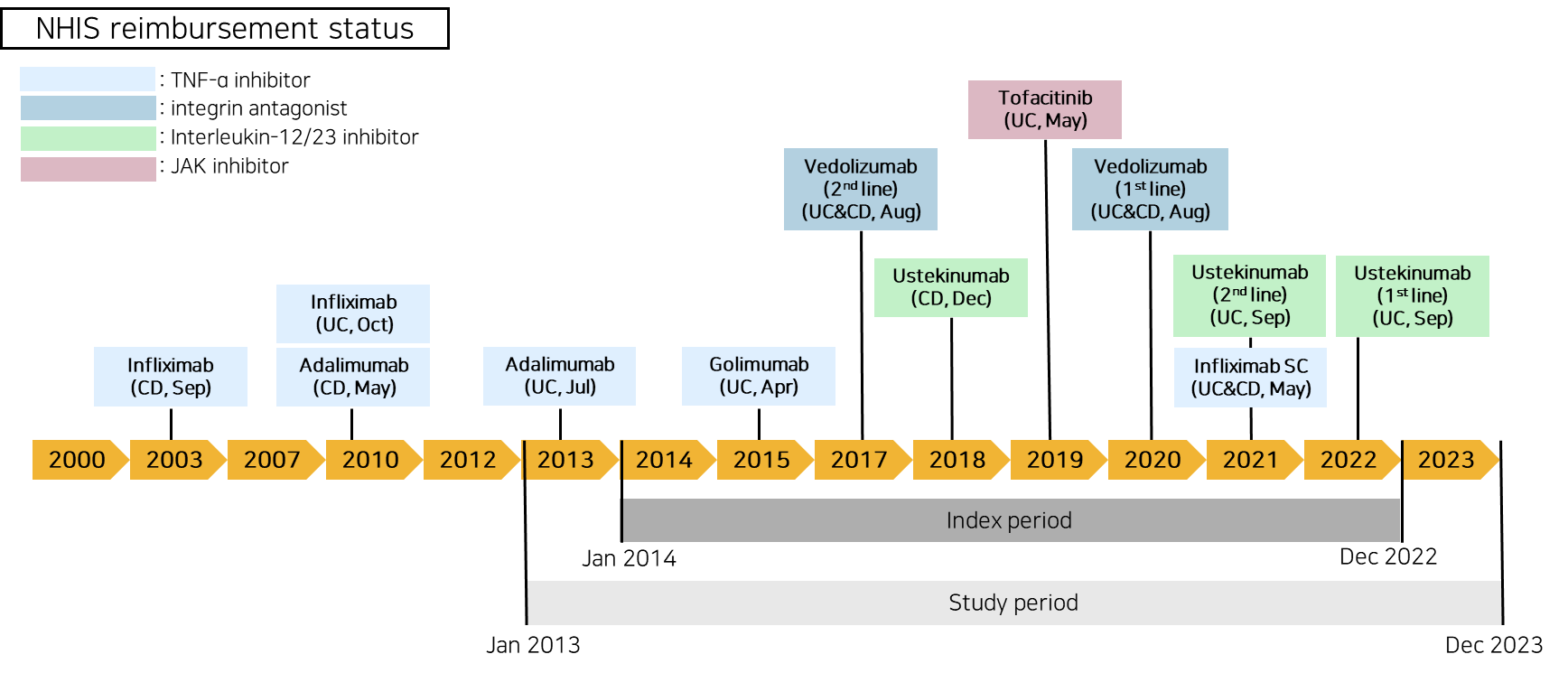


**Abbreviations:** TNF, tumor necrosis factor; JAK, Janus kinase; UC, ulcerative colitis; CD, Crohn’s disease; SC, subcutaneous

**Supplementary Figure S2.** Study design diagram

**
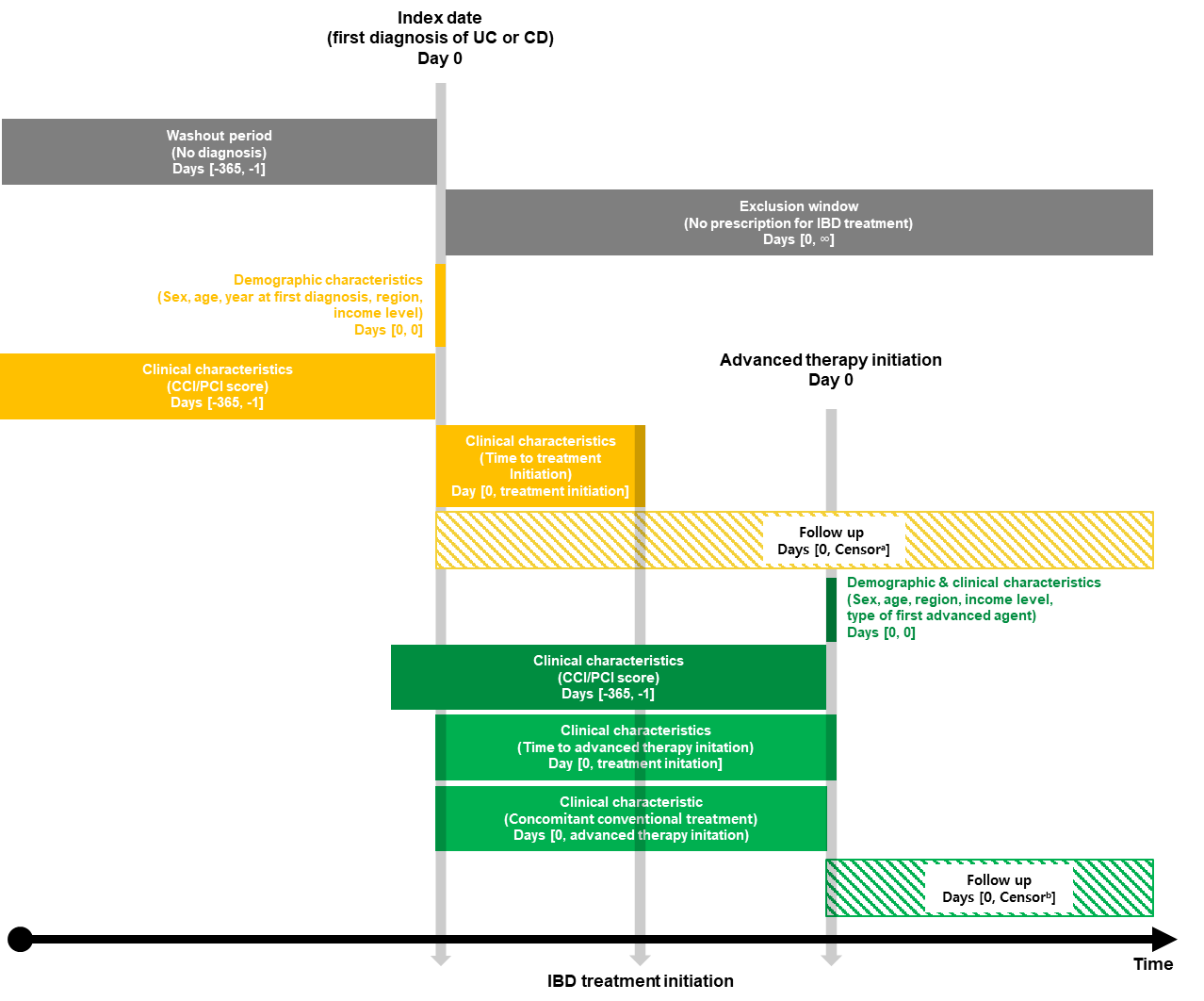
**

**Abbreviations:** IBD, inflammatory bowel disease; UC, ulcerative colitis; CD, Crohn’s disease; CCI, Charlson Comorbidity Index; PCI, Pediatric Comorbidity Index

^a^ For demonstrating overall treatment sequences, patients were followed from their first diagnosis and censored at death, the study end date, or total colectomy (for UC only), whichever occurred first.

^b^ For estimating non-persistence rates and investigating switching patterns, patients were followed from their first diagnosis and censored at death, the study end date, total colectomy (for UC only), 2-year after index date, discontinuation, or switching, whichever occurred first.

**Supplementary Table S1.** List of reimbursed IBD-specific medications

|  |  | **UC** | **CD** |
| --- | --- | --- | --- |
| **Conventional treatment** | |  |  |
| 5-aminosalicylates (5-ASA)^a^ | Mesalamine | ✓ | ✓ |
|  | Balsalazide | ✓ |  |
|  | Sulfasalazine | ✓ |  |
| Corticosteroids (CS)^a^ | Prednisolone | ✓ | ✓ |
|  | Methylprednisolone | ✓ | ✓ |
|  | Hydrocortisone | ✓ | ✓ |
|  | Budesonide |  | ✓ |
| Immunomodulators (IM)^a^ | Azathioprine | ✓ | ✓ |
|  | Mercaptopurine | ✓ | ✓ |
|  | Methotrexate | ✓ | ✓ |
|  | Cyclosporine | ✓ |  |
| **Advanced treatment** | |  |  |
| Tumor necrosis factor (TNF) -alpha inhibitor | Infliximab^a^ | ✓ | ✓ |
|  | Adalimumab^b^ | ✓ | ✓ |
|  | Golimumab | ✓ |  |
| Integrin antagonist | Vedolizumab | ✓ | ✓ |
| Interleukin-12/23 (IL-12/23) inhibitor | Ustekinumab | ✓ | ✓ |
| Janus kinase (JAK) inhibitor | Tofacitinib | ✓ |  |

**Abbreviations:** UC, ulcerative colitis; CD, Crohn’s disease

^a^ Approved for UC/CD patients aged <19 years

^b^ Approved for only CD patients aged <19 years

**Supplementary Table S2.** Reimbursement criteria for advanced therapy

| **IBD subtype** | **Criteria** |
| --- | --- |
| UC | - Patients with moderately to severely active UC who have shown an inadequate response to, intolerance of, or contraindications to conventional therapies, including corticosteroids, 6-mercaptopurine, or azathioprine.   - Moderately to severely active UC is defined as a Mayo score of 6–12 with an endoscopic subscore ≥2. For pediatric patients (≤18 years of age), eligibility is based on a diagnosis of UC and PUCAI >45. |
| CD | - Patients with moderately to severely active CD who have shown an inadequate response to, intolerance of, or contraindications to conventional therapy (at least two agents, including corticosteroids and/or immunomodulators), defined as:   - CDAI ≥220 in adults, or   - PCDAI ≥30 in pediatric patients. |
| CD (fistula) | - Patients with fistulizing CD who have shown an inadequate response to conventional treatment (at least two treatment modalities, such as antibiotics, drainage procedures, and/or immunomodulators). |

**Abbreviations:** UC, ulcerative colitis; CD, Crohn’s disease; SC, subcutaneous; PUCAI, Pediatric Ulcerative Colitis Activity Index; CDAI, Crohn’s Disease Activity Index

**Supplementary Table S3.** Temporal changes in IBD therapy since the first diagnosis

(A) Pediatrics with UC

| **Period^a^** | **Before** | **After** | **Number** | **(%)^b^** |
| --- | --- | --- | --- | --- |
| 1 | Conventional treatment | Conventional treatment | 1238 | 65.8 |
| 1 | Conventional treatment | Infliximab | 129 | 6.9 |
| 1 | Conventional treatment | None | 273 | 14.5 |
| 1 | Infliximab | Conventional treatment | 1 | 0.1 |
| 1 | Infliximab | Infliximab | 36 | 1.9 |
| 1 | Infliximab | None | 1 | 0.1 |
| 1 | None | Conventional treatment | 132 | 7.0 |
| 1 | None | Infliximab | 26 | 1.4 |
| 1 | None | None | 46 | 2.4 |
| 2 | Conventional treatment | Conventional treatment | 1051 | 55.8 |
| 2 | Conventional treatment | Infliximab | 72 | 3.8 |
| 2 | Conventional treatment | None | 246 | 13.1 |
| 2 | Conventional treatment | Censored | 2 | 0.1 |
| 2 | Infliximab | Conventional treatment | 10 | 0.5 |
| 2 | Infliximab | Infliximab | 173 | 9.2 |
| 2 | Infliximab | None | 8 | 0.4 |
| 2 | None | Conventional treatment | 143 | 7.4 |
| 2 | None | Infliximab | 10 | 0.5 |
| 2 | None | None | 167 | 8.9 |
| 3 | Conventional treatment | Conventional treatment | 912 | 48.5 |
| 3 | Conventional treatment | Infliximab | 39 | 2.1 |
| 3 | Conventional treatment | None | 192 | 10.2 |
| 3 | Conventional treatment | Censored | 61 | 3.2 |
| 3 | Infliximab | Conventional treatment | 13 | 0.7 |
| 3 | Infliximab | Infliximab | 206 | 10.9 |
| 3 | Infliximab | None | 18 | 1.0 |
| 3 | Infliximab | Censored | 18 | 1.0 |
| 3 | None | Conventional treatment | 141 | 7.5 |
| 3 | None | Infliximab | 11 | 0.6 |
| 3 | None | None | 215 | 11.4 |
| 3 | None | Censored | 54 | 2.9 |
| 3 | Censored | Censored | 2 | 0.1 |
| 4 | Conventional treatment | Conventional treatment | 768 | 40.8 |
| 4 | Conventional treatment | Infliximab | 32 | 1.7 |
| 4 | Conventional treatment | None | 194 | 10.3 |
| 4 | Conventional treatment | Censored | 72 | 3.8 |
| 4 | Infliximab | Conventional treatment | 12 | 0.6 |
| 4 | Infliximab | Infliximab | 207 | 11.0 |
| 4 | Infliximab | None | 20 | 1.1 |
| 4 | Infliximab | Censored | 17 | 0.9 |
| 4 | None | Conventional treatment | 111 | 5.9 |
| 4 | None | Infliximab | 5 | 0.3 |
| 4 | None | None | 254 | 13.5 |
| 4 | None | Censored | 55 | 2.9 |
| 4 | Censored | Censored | 135 | 7.2 |
| 5 | Conventional treatment | Conventional treatment | 643 | 34.2 |
| 5 | Conventional treatment | Infliximab | 18 | 1.0 |
| 5 | Conventional treatment | None | 169 | 9.0 |
| 5 | Conventional treatment | Censored | 61 | 3.2 |
| 5 | Infliximab | Conventional treatment | 16 | 0.9 |
| 5 | Infliximab | Infliximab | 200 | 10.6 |
| 5 | Infliximab | None | 11 | 0.6 |
| 5 | Infliximab | Censored | 17 | 0.9 |
| 5 | None | Conventional treatment | 143 | 7.6 |
| 5 | None | Infliximab | 2 | 0.1 |
| 5 | None | None | 264 | 14.0 |
| 5 | None | Censored | 59 | 3.1 |
| 5 | Censored | Censored | 279 | 14.8 |

**Abbreviations:** IBD, inflammatory bowel disease; UC, ulcerative colitis

^a^1: 0 to 6 months; 2: 6 to 12 months; 3: 12 to 18 months; 4: 24 to 30 months; 5: 30 to 36 months

^b^denominator: total number of treatment patterns in a specific time period; numerator: number of each pattern in a specific time period

(B) Pediatrics with CD

| **Period^a^** | **Before** | **After** | **Number** | **(%)^b^** |
| --- | --- | --- | --- | --- |
| 1 | Conventional treatment | Conventional treatment | 2035 | 42.9 |
| 1 | Conventional treatment | Infliximab | 832 | 17.5 |
| 1 | Conventional treatment | Adalimumab | 220 | 4.6 |
| 1 | Conventional treatment | None | 344 | 7.2 |
| 1 | Infliximab | Conventional treatment | 5 | 0.1 |
| 1 | Infliximab | Infliximab | 129 | 2.7 |
| 1 | Infliximab | Adalimumab | 1 | 0.0 |
| 1 | Infliximab | None | 4 | 0.1 |
| 1 | Adalimumab | Conventional treatment | 2 | 0.0 |
| 1 | Adalimumab | Infliximab | 3 | 0.1 |
| 1 | Adalimumab | Adalimumab | 25 | 0.5 |
| 1 | Adalimumab | None | 1 | 0.0 |
| 1 | None | Conventional treatment | 633 | 13.3 |
| 1 | None | Infliximab | 331 | 7.0 |
| 1 | None | Adalimumab | 55 | 1.2 |
| 1 | None | None | 127 | 2.7 |
| 2 | Conventional treatment | Conventional treatment | 1922 | 45.0 |
| 2 | Conventional treatment | Infliximab | 300 | 6.3 |
| 2 | Conventional treatment | Adalimumab | 99 | 2.1 |
| 2 | Conventional treatment | None | 353 | 7.4 |
| 2 | Conventional treatment | Censored | 1 | 0.0 |
| 2 | Infliximab | Conventional treatment | 19 | 0.4 |
| 2 | Infliximab | Infliximab | 1237 | 26.1 |
| 2 | Infliximab | Adalimumab | 14 | 0.3 |
| 2 | Infliximab | None | 25 | 0.5 |
| 2 | Adalimumab | Conventional treatment | 10 | 0.2 |
| 2 | Adalimumab | Infliximab | 6 | 0.1 |
| 2 | Adalimumab | Adalimumab | 272 | 5.7 |
| 2 | Adalimumab | None | 13 | 0.3 |
| 2 | None | Conventional treatment | 170 | 3.6 |
| 2 | None | Infliximab | 43 | 0.9 |
| 2 | None | Adalimumab | 14 | 0.3 |
| 2 | None | None | 248 | 5.2 |
| 2 | None | Censored | 1 | 0.0 |
| 3 | Conventional treatment | Conventional treatment | 1221 | 25.7 |
| 3 | Conventional treatment | Infliximab | 43 | 0.9 |
| 3 | Conventional treatment | Adalimumab | 24 | 0.5 |
| 3 | Conventional treatment | None | 286 | 6.0 |
| 3 | Conventional treatment | Censored | 116 | 2.4 |
| 3 | Infliximab | Conventional treatment | 15 | 0.3 |
| 3 | Infliximab | Infliximab | 1316 | 27.7 |
| 3 | Infliximab | Adalimumab | 25 | 0.5 |
| 3 | Infliximab | None | 67 | 1.4 |
| 3 | Infliximab | Censored | 123 | 2.6 |
| 3 | Adalimumab | Conventional treatment | 13 | 0.3 |
| 3 | Adalimumab | Infliximab | 2 | 0.0 |
| 3 | Adalimumab | Adalimumab | 369 | 7.8 |
| 3 | Adalimumab | None | 23 | 0.5 |
| 3 | Adalimumab | Censored | 25 | 0.5 |
| 3 | None | Conventional treatment | 164 | 3.5 |
| 3 | None | Infliximab | 22 | 0.5 |
| 3 | None | Adalimumab | 7 | 0.1 |
| 3 | None | None | 330 | 7.0 |
| 3 | None | Censored | 192 | 4.0 |
| 3 | Censored | Censored | 364 | 7.7 |
| 4 | Conventional treatment | Conventional treatment | 982 | 20.7 |
| 4 | Conventional treatment | Infliximab | 47 | 1.0 |
| 4 | Conventional treatment | Adalimumab | 21 | 0.4 |
| 4 | Conventional treatment | None | 243 | 5.1 |
| 4 | Conventional treatment | Censored | 120 | 2.5 |
| 4 | Infliximab | Conventional treatment | 24 | 0.5 |
| 4 | Infliximab | Infliximab | 1117 | 23.5 |
| 4 | Infliximab | Adalimumab | 21 | 0.4 |
| 4 | Infliximab | None | 74 | 1.6 |
| 4 | Infliximab | Censored | 147 | 3.1 |
| 4 | Adalimumab | Conventional treatment | 9 | 0.2 |
| 4 | Adalimumab | Infliximab | 4 | 0.1 |
| 4 | Adalimumab | Adalimumab | 368 | 7.8 |
| 4 | Adalimumab | None | 24 | 0.5 |
| 4 | Adalimumab | Censored | 20 | 0.4 |
| 4 | None | Conventional treatment | 164 | 3.5 |
| 4 | None | Infliximab | 12 | 0.3 |
| 4 | None | Adalimumab | 10 | 0.2 |
| 4 | None | None | 331 | 7.0 |
| 4 | None | Censored | 189 | 4.0 |
| 4 | Censored | Censored | 820 | 17.3 |
| 5 | Conventional treatment | Conventional treatment | 832 | 17.5 |
| 5 | Conventional treatment | Infliximab | 21 | 0.4 |
| 5 | Conventional treatment | Adalimumab | 21 | 0.4 |
| 5 | Conventional treatment | None | 202 | 4.3 |
| 5 | Conventional treatment | Censored | 103 | 2.2 |
| 5 | Infliximab | Conventional treatment | 18 | 0.4 |
| 5 | Infliximab | Infliximab | 960 | 20.2 |
| 5 | Infliximab | Adalimumab | 12 | 0.3 |
| 5 | Infliximab | None | 67 | 1.4 |
| 5 | Infliximab | Censored | 123 | 2.6 |
| 5 | Adalimumab | Conventional treatment | 15 | 0.3 |
| 5 | Adalimumab | Infliximab | 3 | 0.1 |
| 5 | Adalimumab | Adalimumab | 355 | 7.5 |
| 5 | Adalimumab | None | 30 | 0.6 |
| 5 | Adalimumab | Censored | 17 | 0.4 |
| 5 | None | Conventional treatment | 154 | 3.2 |
| 5 | None | Infliximab | 20 | 0.4 |
| 5 | None | Adalimumab | 7 | 0.1 |
| 5 | None | None | 341 | 7.2 |
| 5 | None | Censored | 150 | 3.2 |
| 5 | Censored | Censored | 1296 | 27.3 |

**Abbreviations:** IBD, inflammatory bowel disease; CD, Crohn’s disease

^a^1: 0 to 6 months; 2: 6 to 12 months; 3: 12 to 18 months; 4: 24 to 30 months; 5: 30 to 36 months

^b^denominator: total number of treatment patterns in a specific time period; numerator: number of each pattern in a specific time period

(C) Adults with UC

| **Period^a^** | **Before** | **After** | **Number** | **(%)^b^** |
| --- | --- | --- | --- | --- |
| 1 | Conventional treatment | Conventional treatment | 24783 | 61.5 |
| 1 | Conventional treatment | TNF-alpha inhibitors | 476 | 1.2 |
| 1 | Conventional treatment | Integrin receptor antagonists | 71 | 0.2 |
| 1 | Conventional treatment | IL-12/23 inhibitors | 7 | 0.0 |
| 1 | Conventional treatment | JAK inhibitors | 39 | 0.1 |
| 1 | Conventional treatment | None | 10226 | 25.4 |
| 1 | Conventional treatment | Censored | 113 | 0.3 |
| 1 | TNF-alpha inhibitors | Conventional treatment | 18 | 0.0 |
| 1 | TNF-alpha inhibitors | TNF-alpha inhibitors | 159 | 0.4 |
| 1 | TNF-alpha inhibitors | Integrin receptor antagonists | 1 | 0.0 |
| 1 | TNF-alpha inhibitors | IL-12/23 inhibitors | 1 | 0.0 |
| 1 | TNF-alpha inhibitors | JAK inhibitors | 1 | 0.0 |
| 1 | TNF-alpha inhibitors | None | 23 | 0.1 |
| 1 | TNF-alpha inhibitors | Censored | 5 | 0.0 |
| 1 | Integrin receptor antagonists | Conventional treatment | 1 | 0.0 |
| 1 | Integrin receptor antagonists | TNF-alpha inhibitors | 1 | 0.0 |
| 1 | Integrin receptor antagonists | Integrin receptor antagonists | 1 | 0.0 |
| 1 | Integrin receptor antagonists | IL-12/23 inhibitors | 1 | 0.0 |
| 1 | Integrin receptor antagonists | JAK inhibitors | 1 | 0.0 |
| 1 | Integrin receptor antagonists | None | 1 | 0.0 |
| 1 | Integrin receptor antagonists | Integrin receptor antagonists | 1 | 0.0 |
| 1 | JAK inhibitors | TNF-alpha inhibitors | 1 | 0.0 |
| 1 | JAK inhibitors | JAK inhibitors | 3 | 0.0 |
| 1 | None | Conventional treatment | 2563 | 6.4 |
| 1 | None | TNF-alpha inhibitors | 66 | 0.2 |
| 1 | None | Integrin receptor antagonists | 11 | 0.0 |
| 1 | None | IL-12/23 inhibitors | 1 | 0.0 |
| 1 | None | JAK inhibitors | 4 | 0.0 |
| 1 | None | None | 1700 | 4.2 |
| 1 | None | Censored | 9 | 0.0 |
| 2 | Conventional treatment | Conventional treatment | 20973 | 52.1 |
| 2 | Conventional treatment | TNF-alpha inhibitors | 368 | 0.9 |
| 2 | Conventional treatment | Integrin receptor antagonists | 92 | 0.2 |
| 2 | Conventional treatment | IL-12/23 inhibitors | 17 | 0.0 |
| 2 | Conventional treatment | JAK inhibitors | 35 | 0.1 |
| 2 | Conventional treatment | None | 5840 | 14.5 |
| 2 | Conventional treatment | Censored | 40 | 0.1 |
| 2 | TNF-alpha inhibitors | Conventional treatment | 74 | 0.2 |
| 2 | TNF-alpha inhibitors | TNF-alpha inhibitors | 553 | 1.4 |
| 2 | TNF-alpha inhibitors | Integrin receptor antagonists | 10 | 0.0 |
| 2 | TNF-alpha inhibitors | IL-12/23 inhibitors | 2 | 0.0 |
| 2 | TNF-alpha inhibitors | JAK inhibitors | 13 | 0.0 |
| 2 | TNF-alpha inhibitors | None | 50 | 0.1 |
| 2 | TNF-alpha inhibitors | Censored | 1 | 0.0 |
| 2 | Integrin receptor antagonists | Conventional treatment | 7 | 0.0 |
| 2 | Integrin receptor antagonists | TNF-alpha inhibitors | 5 | 0.0 |
| 2 | Integrin receptor antagonists | Integrin receptor antagonists | 56 | 0.1 |
| 2 | Integrin receptor antagonists | IL-12/23 inhibitors | 2 | 0.0 |
| 2 | Integrin receptor antagonists | JAK inhibitors | 4 | 0.0 |
| 2 | Integrin receptor antagonists | None | 10 | 0.0 |
| 2 | IL-12/23 inhibitors | IL-12/23 inhibitors | 9 | 0.0 |
| 2 | IL-12/23 inhibitors | None | 2 | 0.0 |
| 2 | JAK inhibitors | Conventional treatment | 3 | 0.0 |
| 2 | JAK inhibitors | TNF-alpha inhibitors | 3 | 0.0 |
| 2 | JAK inhibitors | Integrin receptor antagonists | 2 | 0.0 |
| 2 | JAK inhibitors | JAK inhibitors | 36 | 0.1 |
| 2 | JAK inhibitors | None | 3 | 0.0 |
| 2 | None | Conventional treatment | 3390 | 8.4 |
| 2 | None | TNF-alpha inhibitors | 58 | 0.1 |
| 2 | None | Integrin receptor antagonists | 9 | 0.0 |
| 2 | None | IL-12/23 inhibitors | 2 | 0.0 |
| 2 | None | JAK inhibitors | 4 | 0.0 |
| 2 | None | None | 8460 | 21.0 |
| 2 | None | Censored | 27 | 0.1 |
| 2 | Censored | Censored | 128 | 0.3 |
| 3 | Conventional treatment | Conventional treatment | 17980 | 44.6 |
| 3 | Conventional treatment | TNF-alpha inhibitors | 274 | 0.7 |
| 3 | Conventional treatment | Integrin receptor antagonists | 89 | 0.2 |
| 3 | Conventional treatment | IL-12/23 inhibitors | 23 | 0.1 |
| 3 | Conventional treatment | JAK inhibitors | 33 | 0.1 |
| 3 | Conventional treatment | None | 4905 | 12.2 |
| 3 | Conventional treatment | Censored | 1143 | 2.8 |
| 3 | TNF-alpha inhibitors | Conventional treatment | 79 | 0.2 |
| 3 | TNF-alpha inhibitors | TNF-alpha inhibitors | 780 | 1.9 |
| 3 | TNF-alpha inhibitors | Integrin receptor antagonists | 10 | 0.0 |
| 3 | TNF-alpha inhibitors | IL-12/23 inhibitors | 7 | 0.0 |
| 3 | TNF-alpha inhibitors | JAK inhibitors | 23 | 0.1 |
| 3 | TNF-alpha inhibitors | None | 57 | 0.1 |
| 3 | TNF-alpha inhibitors | Censored | 31 | 0.1 |
| 3 | Integrin receptor antagonists | Conventional treatment | 6 | 0.0 |
| 3 | Integrin receptor antagonists | TNF-alpha inhibitors | 5 | 0.0 |
| 3 | Integrin receptor antagonists | Integrin receptor antagonists | 124 | 0.3 |
| 3 | Integrin receptor antagonists | IL-12/23 inhibitors | 6 | 0.0 |
| 3 | Integrin receptor antagonists | JAK inhibitors | 3 | 0.0 |
| 3 | Integrin receptor antagonists | None | 12 | 0.0 |
| 3 | Integrin receptor antagonists | Censored | 13 | 0.0 |
| 3 | IL-12/23 inhibitors | TNF-alpha inhibitors | 1 | 0.0 |
| 3 | IL-12/23 inhibitors | IL-12/23 inhibitors | 14 | 0.0 |
| 3 | IL-12/23 inhibitors | None | 6 | 0.0 |
| 3 | IL-12/23 inhibitors | Censored | 11 | 0.0 |
| 3 | JAK inhibitors | Conventional treatment | 4 | 0.0 |
| 3 | JAK inhibitors | TNF-alpha inhibitors | 2 | 0.0 |
| 3 | JAK inhibitors | Integrin receptor antagonists | 1 | 0.0 |
| 3 | JAK inhibitors | JAK inhibitors | 65 | 0.2 |
| 3 | JAK inhibitors | None | 14 | 0.0 |
| 3 | JAK inhibitors | Censored | 6 | 0.0 |
| 3 | None | Conventional treatment | 3521 | 8.7 |
| 3 | None | TNF-alpha inhibitors | 50 | 0.1 |
| 3 | None | Integrin receptor antagonists | 7 | 0.0 |
| 3 | None | IL-12/23 inhibitors | 4 | 0.0 |
| 3 | None | JAK inhibitors | 4 | 0.0 |
| 3 | None | None | 9794 | 24.3 |
| 3 | None | Censored | 985 | 2.4 |
| 3 | Censored | Censored | 196 | 0.5 |
| 4 | Conventional treatment | Conventional treatment | 15929 | 39.5 |
| 4 | Conventional treatment | TNF-alpha inhibitors | 204 | 0.5 |
| 4 | Conventional treatment | Integrin receptor antagonists | 75 | 0.2 |
| 4 | Conventional treatment | IL-12/23 inhibitors | 22 | 0.1 |
| 4 | Conventional treatment | JAK inhibitors | 28 | 0.1 |
| 4 | Conventional treatment | None | 4135 | 10.3 |
| 4 | Conventional treatment | Censored | 1197 | 3.0 |
| 4 | TNF-alpha inhibitors | Conventional treatment | 82 | 0.2 |
| 4 | TNF-alpha inhibitors | TNF-alpha inhibitors | 876 | 2.2 |
| 4 | TNF-alpha inhibitors | Integrin receptor antagonists | 13 | 0.0 |
| 4 | TNF-alpha inhibitors | IL-12/23 inhibitors | 9 | 0.0 |
| 4 | TNF-alpha inhibitors | JAK inhibitors | 15 | 0.0 |
| 4 | TNF-alpha inhibitors | None | 81 | 0.2 |
| 4 | TNF-alpha inhibitors | Censored | 36 | 0.1 |
| 4 | Integrin receptor antagonists | Conventional treatment | 15 | 0.0 |
| 4 | Integrin receptor antagonists | TNF-alpha inhibitors | 7 | 0.0 |
| 4 | Integrin receptor antagonists | Integrin receptor antagonists | 172 | 0.4 |
| 4 | Integrin receptor antagonists | IL-12/23 inhibitors | 5 | 0.0 |
| 4 | Integrin receptor antagonists | JAK inhibitors | 2 | 0.0 |
| 4 | Integrin receptor antagonists | None | 12 | 0.0 |
| 4 | Integrin receptor antagonists | Censored | 17 | 0.0 |
| 4 | IL-12/23 inhibitors | Conventional treatment | 1 | 0.0 |
| 4 | IL-12/23 inhibitors | Integrin receptor antagonists | 1 | 0.0 |
| 4 | IL-12/23 inhibitors | IL-12/23 inhibitors | 23 | 0.1 |
| 4 | IL-12/23 inhibitors | JAK inhibitors | 2 | 0.0 |
| 4 | IL-12/23 inhibitors | None | 6 | 0.0 |
| 4 | IL-12/23 inhibitors | Censored | 22 | 0.1 |
| 4 | JAK inhibitors | Conventional treatment | 5 | 0.0 |
| 4 | JAK inhibitors | TNF-alpha inhibitors | 9 | 0.0 |
| 4 | JAK inhibitors | Integrin receptor antagonists | 4 | 0.0 |
| 4 | JAK inhibitors | IL-12/23 inhibitors | 3 | 0.0 |
| 4 | JAK inhibitors | JAK inhibitors | 90 | 0.2 |
| 4 | JAK inhibitors | None | 7 | 0.0 |
| 4 | JAK inhibitors | Censored | 10 | 0.0 |
| 4 | None | Conventional treatment | 3317 | 8.2 |
| 4 | None | TNF-alpha inhibitors | 41 | 0.1 |
| 4 | None | Integrin receptor antagonists | 11 | 0.0 |
| 4 | None | IL-12/23 inhibitors | 9 | 0.0 |
| 4 | None | JAK inhibitors | 4 | 0.0 |
| 4 | None | None | 10231 | 25.4 |
| 4 | None | Censored | 1175 | 2.9 |
| 4 | Censored | Censored | 2385 | 5.9 |
| 5 | Conventional treatment | Conventional treatment | 14214 | 35.3 |
| 5 | Conventional treatment | TNF-alpha inhibitors | 149 | 0.4 |
| 5 | Conventional treatment | Integrin receptor antagonists | 59 | 0.1 |
| 5 | Conventional treatment | IL-12/23 inhibitors | 26 | 0.1 |
| 5 | Conventional treatment | JAK inhibitors | 29 | 0.1 |
| 5 | Conventional treatment | None | 3812 | 9.5 |
| 5 | Conventional treatment | Censored | 1060 | 2.6 |
| 5 | TNF-alpha inhibitors | Conventional treatment | 79 | 0.2 |
| 5 | TNF-alpha inhibitors | TNF-alpha inhibitors | 918 | 2.3 |
| 5 | TNF-alpha inhibitors | Integrin receptor antagonists | 9 | 0.0 |
| 5 | TNF-alpha inhibitors | IL-12/23 inhibitors | 10 | 0.0 |
| 5 | TNF-alpha inhibitors | JAK inhibitors | 18 | 0.0 |
| 5 | TNF-alpha inhibitors | None | 65 | 0.2 |
| 5 | TNF-alpha inhibitors | Censored | 38 | 0.1 |
| 5 | Integrin receptor antagonists | Conventional treatment | 8 | 0.0 |
| 5 | Integrin receptor antagonists | TNF-alpha inhibitors | 4 | 0.0 |
| 5 | Integrin receptor antagonists | Integrin receptor antagonists | 189 | 0.5 |
| 5 | Integrin receptor antagonists | IL-12/23 inhibitors | 5 | 0.0 |
| 5 | Integrin receptor antagonists | JAK inhibitors | 4 | 0.0 |
| 5 | Integrin receptor antagonists | None | 30 | 0.1 |
| 5 | Integrin receptor antagonists | Censored | 36 | 0.1 |
| 5 | IL-12/23 inhibitors | TNF-alpha inhibitors | 3 | 0.0 |
| 5 | IL-12/23 inhibitors | IL-12/23 inhibitors | 33 | 0.1 |
| 5 | IL-12/23 inhibitors | JAK inhibitors | 3 | 0.0 |
| 5 | IL-12/23 inhibitors | None | 11 | 0.0 |
| 5 | IL-12/23 inhibitors | Censored | 21 | 0.1 |
| 5 | JAK inhibitors | Conventional treatment | 2 | 0.0 |
| 5 | JAK inhibitors | TNF-alpha inhibitors | 3 | 0.0 |
| 5 | JAK inhibitors | Integrin receptor antagonists | 3 | 0.0 |
| 5 | JAK inhibitors | JAK inhibitors | 119 | 0.3 |
| 5 | JAK inhibitors | None | 8 | 0.0 |
| 5 | JAK inhibitors | Censored | 6 | 0.0 |
| 5 | None | Conventional treatment | 3015 | 7.5 |
| 5 | None | TNF-alpha inhibitors | 39 | 0.1 |
| 5 | None | Integrin receptor antagonists | 8 | 0.0 |
| 5 | None | IL-12/23 inhibitors | 2 | 0.0 |
| 5 | None | JAK inhibitors | 3 | 0.0 |
| 5 | None | None | 10219 | 25.4 |
| 5 | None | Censored | 1186 | 2.9 |
| 5 | Censored | Censored | 4842 | 12.0 |
| 6 | Conventional treatment | Conventional treatment | 12719 | 31.6 |
| 6 | Conventional treatment | TNF-alpha inhibitors | 137 | 0.3 |
| 6 | Conventional treatment | Integrin receptor antagonists | 61 | 0.2 |
| 6 | Conventional treatment | IL-12/23 inhibitors | 20 | 0.0 |
| 6 | Conventional treatment | JAK inhibitors | 22 | 0.1 |
| 6 | Conventional treatment | None | 3229 | 8.0 |
| 6 | Conventional treatment | Censored | 1130 | 2.8 |
| 6 | TNF-alpha inhibitors | Conventional treatment | 57 | 0.1 |
| 6 | TNF-alpha inhibitors | TNF-alpha inhibitors | 940 | 2.3 |
| 6 | TNF-alpha inhibitors | Integrin receptor antagonists | 5 | 0.0 |
| 6 | TNF-alpha inhibitors | IL-12/23 inhibitors | 7 | 0.0 |
| 6 | TNF-alpha inhibitors | JAK inhibitors | 11 | 0.0 |
| 6 | TNF-alpha inhibitors | None | 58 | 0.1 |
| 6 | TNF-alpha inhibitors | Censored | 38 | 0.1 |
| 6 | Integrin receptor antagonists | Conventional treatment | 10 | 0.0 |
| 6 | Integrin receptor antagonists | TNF-alpha inhibitors | 4 | 0.0 |
| 6 | Integrin receptor antagonists | Integrin receptor antagonists | 179 | 0.4 |
| 6 | Integrin receptor antagonists | IL-12/23 inhibitors | 3 | 0.0 |
| 6 | Integrin receptor antagonists | JAK inhibitors | 3 | 0.0 |
| 6 | Integrin receptor antagonists | None | 31 | 0.1 |
| 6 | Integrin receptor antagonists | Censored | 38 | 0.1 |
| 6 | IL-12/23 inhibitors | IL-12/23 inhibitors | 36 | 0.1 |
| 6 | IL-12/23 inhibitors | None | 11 | 0.0 |
| 6 | IL-12/23 inhibitors | Censored | 29 | 0.1 |
| 6 | JAK inhibitors | Conventional treatment | 12 | 0.0 |
| 6 | JAK inhibitors | TNF-alpha inhibitors | 3 | 0.0 |
| 6 | JAK inhibitors | IL-12/23 inhibitors | 1 | 0.0 |
| 6 | JAK inhibitors | JAK inhibitors | 139 | 0.3 |
| 6 | JAK inhibitors | None | 13 | 0.0 |
| 6 | JAK inhibitors | Censored | 8 | 0.0 |
| 6 | None | Conventional treatment | 2837 | 7.0 |
| 6 | None | TNF-alpha inhibitors | 35 | 0.1 |
| 6 | None | Integrin receptor antagonists | 10 | 0.0 |
| 6 | None | IL-12/23 inhibitors | 4 | 0.0 |
| 6 | None | JAK inhibitors | 7 | 0.0 |
| 6 | None | None | 10021 | 24.9 |
| 6 | None | Censored | 1231 | 3.1 |
| 6 | Censored | Censored | 7189 | 17.8 |

**Abbreviations:** IBD, inflammatory bowel disease; UC, ulcerative colitis; TNF, tumor necrosis factor; IL, interleukin; JAK, Janus kinase

^a^1: 0 to 6 months; 2: 6 to 12 months; 3: 12 to 18 months; 4: 24 to 30 months; 5: 30 to 36 months

^b^denominator: total number of treatment patterns in a specific time period; numerator: number of each pattern in a specific time period

(D) Adults with CD

| **Period^a^** | **Before** | **After** | **Number** | **(%)^b^** |
| --- | --- | --- | --- | --- |
| 1 | Conventional treatment | Conventional treatment | 6947 | 52.4 |
| 1 | Conventional treatment | TNF-alpha inhibitors | 987 | 7.4 |
| 1 | Conventional treatment | Integrin receptor antagonists | 33 | 0.2 |
| 1 | Conventional treatment | IL-12/23 inhibitors | 107 | 0.8 |
| 1 | Conventional treatment | None | 1941 | 14.6 |
| 1 | Conventional treatment | Censored | 57 | 0.4 |
| 1 | TNF-alpha inhibitors | Conventional treatment | 23 | 0.2 |
| 1 | TNF-alpha inhibitors | TNF-alpha inhibitors | 202 | 1.5 |
| 1 | TNF-alpha inhibitors | None | 18 | 0.1 |
| 1 | TNF-alpha inhibitors | Censored | 1 | 0.0 |
| 1 | Integrin receptor antagonists | Integrin receptor antagonists | 3 | 0.0 |
| 1 | IL-12/23 inhibitors | IL-12/23 inhibitors | 10 | 0.1 |
| 1 | None | Conventional treatment | 1774 | 13.4 |
| 1 | None | TNF-alpha inhibitors | 363 | 2.7 |
| 1 | None | Integrin receptor antagonists | 10 | 0.1 |
| 1 | None | IL-12/23 inhibitors | 23 | 0.2 |
| 1 | None | None | 755 | 5.7 |
| 1 | None | Censored | 10 | 0.1 |
| 2 | Conventional treatment | Conventional treatment | 6493 | 49.0 |
| 2 | Conventional treatment | TNF-alpha inhibitors | 540 | 4.1 |
| 2 | Conventional treatment | Integrin receptor antagonists | 40 | 0.3 |
| 2 | Conventional treatment | IL-12/23 inhibitors | 132 | 1.0 |
| 2 | Conventional treatment | None | 1519 | 11.5 |
| 2 | Conventional treatment | Censored | 20 | 0.2 |
| 2 | TNF-alpha inhibitors | Conventional treatment | 43 | 0.3 |
| 2 | TNF-alpha inhibitors | TNF-alpha inhibitors | 1418 | 10.7 |
| 2 | TNF-alpha inhibitors | Integrin receptor antagonists | 1 | 0.0 |
| 2 | TNF-alpha inhibitors | IL-12/23 inhibitors | 5 | 0.0 |
| 2 | TNF-alpha inhibitors | None | 79 | 0.6 |
| 2 | TNF-alpha inhibitors | Censored | 6 | 0.0 |
| 2 | Integrin receptor antagonists | Conventional treatment | 1 | 0.0 |
| 2 | Integrin receptor antagonists | TNF-alpha inhibitors | 3 | 0.0 |
| 2 | Integrin receptor antagonists | Integrin receptor antagonists | 40 | 0.3 |
| 2 | Integrin receptor antagonists | None | 1 | 0.0 |
| 2 | Integrin receptor antagonists | Censored | 1 | 0.0 |
| 2 | IL-12/23 inhibitors | Conventional treatment | 2 | 0.0 |
| 2 | IL-12/23 inhibitors | TNF-alpha inhibitors | 4 | 0.0 |
| 2 | IL-12/23 inhibitors | Integrin receptor antagonists | 1 | 0.0 |
| 2 | IL-12/23 inhibitors | IL-12/23 inhibitors | 121 | 0.9 |
| 2 | IL-12/23 inhibitors | None | 10 | 0.1 |
| 2 | IL-12/23 inhibitors | Censored | 2 | 0.0 |
| 2 | None | Conventional treatment | 711 | 5.4 |
| 2 | None | TNF-alpha inhibitors | 97 | 0.7 |
| 2 | None | Integrin receptor antagonists | 1 | 0.0 |
| 2 | None | IL-12/23 inhibitors | 7 | 0.1 |
| 2 | None | None | 1885 | 14.2 |
| 2 | None | Censored | 13 | 0.1 |
| 2 | Censored | Censored | 68 | 0.5 |
| 3 | Conventional treatment | Conventional treatment | 5341 | 40.3 |
| 3 | Conventional treatment | TNF-alpha inhibitors | 292 | 2.2 |
| 3 | Conventional treatment | Integrin receptor antagonists | 26 | 0.2 |
| 3 | Conventional treatment | IL-12/23 inhibitors | 75 | 0.6 |
| 3 | Conventional treatment | None | 1208 | 9.1 |
| 3 | Conventional treatment | Censored | 308 | 2.3 |
| 3 | TNF-alpha inhibitors | Conventional treatment | 46 | 0.3 |
| 3 | TNF-alpha inhibitors | TNF-alpha inhibitors | 1823 | 13.7 |
| 3 | TNF-alpha inhibitors | Integrin receptor antagonists | 1 | 0.0 |
| 3 | TNF-alpha inhibitors | IL-12/23 inhibitors | 14 | 0.1 |
| 3 | TNF-alpha inhibitors | None | 110 | 0.8 |
| 3 | TNF-alpha inhibitors | Censored | 68 | 0.5 |
| 3 | Integrin receptor antagonists | Conventional treatment | 1 | 0.0 |
| 3 | Integrin receptor antagonists | TNF-alpha inhibitors | 5 | 0.0 |
| 3 | Integrin receptor antagonists | Integrin receptor antagonists | 64 | 0.5 |
| 3 | Integrin receptor antagonists | IL-12/23 inhibitors | 1 | 0.0 |
| 3 | Integrin receptor antagonists | None | 3 | 0.0 |
| 3 | Integrin receptor antagonists | Censored | 9 | 0.1 |
| 3 | IL-12/23 inhibitors | Conventional treatment | 3 | 0.0 |
| 3 | IL-12/23 inhibitors | TNF-alpha inhibitors | 11 | 0.1 |
| 3 | IL-12/23 inhibitors | Integrin receptor antagonists | 205 | 1.5 |
| 3 | IL-12/23 inhibitors | None | 17 | 0.1 |
| 3 | IL-12/23 inhibitors | Censored | 29 | 0.2 |
| 3 | None | Conventional treatment | 778 | 5.9 |
| 3 | None | TNF-alpha inhibitors | 76 | 0.6 |
| 3 | None | Integrin receptor antagonists | 5 | 0.0 |
| 3 | None | IL-12/23 inhibitors | 6 | 0.0 |
| 3 | None | None | 2330 | 17.6 |
| 3 | None | Censored | 299 | 2.3 |
| 3 | Censored | Censored | 110 | 0.8 |
| 4 | Conventional treatment | Conventional treatment | 4562 | 34.4 |
| 4 | Conventional treatment | TNF-alpha inhibitors | 167 | 1.3 |
| 4 | Conventional treatment | Integrin receptor antagonists | 10 | 0.1 |
| 4 | Conventional treatment | IL-12/23 inhibitors | 44 | 0.3 |
| 4 | Conventional treatment | None | 1056 | 8.0 |
| 4 | Conventional treatment | Censored | 330 | 2.5 |
| 4 | TNF-alpha inhibitors | Conventional treatment | 45 | 0.3 |
| 4 | TNF-alpha inhibitors | TNF-alpha inhibitors | 1946 | 14.7 |
| 4 | TNF-alpha inhibitors | IL-12/23 inhibitors | 20 | 0.2 |
| 4 | TNF-alpha inhibitors | None | 115 | 0.9 |
| 4 | TNF-alpha inhibitors | Censored | 81 | 0.6 |
| 4 | Integrin receptor antagonists | Conventional treatment | 3 | 0.0 |
| 4 | Integrin receptor antagonists | TNF-alpha inhibitors | 9 | 0.1 |
| 4 | Integrin receptor antagonists | Integrin receptor antagonists | 61 | 0.5 |
| 4 | Integrin receptor antagonists | IL-12/23 inhibitors | 5 | 0.0 |
| 4 | Integrin receptor antagonists | None | 7 | 0.1 |
| 4 | Integrin receptor antagonists | Censored | 11 | 0.1 |
| 4 | IL-12/23 inhibitors | TNF-alpha inhibitors | 3 | 0.0 |
| 4 | IL-12/23 inhibitors | Integrin receptor antagonists | 241 | 1.8 |
| 4 | IL-12/23 inhibitors | None | 19 | 0.1 |
| 4 | IL-12/23 inhibitors | Censored | 38 | 0.3 |
| 4 | None | Conventional treatment | 690 | 5.2 |
| 4 | None | TNF-alpha inhibitors | 72 | 0.5 |
| 4 | None | Integrin receptor antagonists | 4 | 0.0 |
| 4 | None | IL-12/23 inhibitors | 7 | 0.1 |
| 4 | None | None | 2533 | 19.1 |
| 4 | None | Censored | 362 | 2.7 |
| 4 | Censored | Censored | 823 | 6.2 |
| 5 | Conventional treatment | Conventional treatment | 3964 | 29.9 |
| 5 | Conventional treatment | TNF-alpha inhibitors | 139 | 1.0 |
| 5 | Conventional treatment | Integrin receptor antagonists | 14 | 0.1 |
| 5 | Conventional treatment | IL-12/23 inhibitors | 33 | 0.2 |
| 5 | Conventional treatment | None | 876 | 6.6 |
| 5 | Conventional treatment | Censored | 274 | 2.1 |
| 5 | TNF-alpha inhibitors | Conventional treatment | 34 | 0.3 |
| 5 | TNF-alpha inhibitors | TNF-alpha inhibitors | 1921 | 14.5 |
| 5 | TNF-alpha inhibitors | Integrin receptor antagonists | 1 | 0.0 |
| 5 | TNF-alpha inhibitors | IL-12/23 inhibitors | 13 | 0.1 |
| 5 | TNF-alpha inhibitors | None | 128 | 1.0 |
| 5 | TNF-alpha inhibitors | Censored | 100 | 0.8 |
| 5 | Integrin receptor antagonists | Conventional treatment | 1 | 0.0 |
| 5 | Integrin receptor antagonists | TNF-alpha inhibitors | 6 | 0.0 |
| 5 | Integrin receptor antagonists | Integrin receptor antagonists | 53 | 0.4 |
| 5 | Integrin receptor antagonists | IL-12/23 inhibitors | 1 | 0.0 |
| 5 | Integrin receptor antagonists | None | 9 | 0.1 |
| 5 | Integrin receptor antagonists | Censored | 5 | 0.0 |
| 5 | IL-12/23 inhibitors | Conventional treatment | 1 | 0.0 |
| 5 | IL-12/23 inhibitors | TNF-alpha inhibitors | 1 | 0.0 |
|  | IL-12/23 inhibitors | Integrin receptor antagonists | 1 | 0.0 |
| 5 | IL-12/23 inhibitors | IL-12/23 inhibitors | 235 | 1.8 |
| 5 | IL-12/23 inhibitors | None | 22 | 0.2 |
| 5 | IL-12/23 inhibitors | Censored | 57 | 0.4 |
| 5 | None | Conventional treatment | 664 | 5.0 |
| 5 | None | TNF-alpha inhibitors | 79 | 0.6 |
| 5 | None | Integrin receptor antagonists | 5 | 0.0 |
| 5 | None | IL-12/23 inhibitors | 11 | 0.1 |
| 5 | None | None | 2618 | 19.7 |
| 5 | None | Censored | 353 | 2.7 |
| 5 | Censored | Censored | 1645 | 12.4 |
| 6 | Conventional treatment | Conventional treatment | 3459 | 26.1 |
| 6 | Conventional treatment | TNF-alpha inhibitors | 112 | 0.8 |
| 6 | Conventional treatment | Integrin receptor antagonists | 6 | 0.0 |
| 6 | Conventional treatment | IL-12/23 inhibitors | 29 | 0.2 |
| 6 | Conventional treatment | None | 777 | 5.9 |
| 6 | Conventional treatment | Censored | 281 | 2.1 |
| 6 | TNF-alpha inhibitors | Conventional treatment | 57 | 0.4 |
| 6 | TNF-alpha inhibitors | TNF-alpha inhibitors | 1848 | 13.9 |
| 6 | TNF-alpha inhibitors | Integrin receptor antagonists | 4 | 0.0 |
| 6 | TNF-alpha inhibitors | IL-12/23 inhibitors | 18 | 0.1 |
| 6 | TNF-alpha inhibitors | None | 96 | 0.7 |
| 6 | TNF-alpha inhibitors | Censored | 123 | 0.9 |
| 6 | Integrin receptor antagonists | TNF-alpha inhibitors | 3 | 0.0 |
| 6 | Integrin receptor antagonists | Integrin receptor antagonists | 52 | 0.4 |
| 6 | Integrin receptor antagonists | IL-12/23 inhibitors | 3 | 0.0 |
| 6 | Integrin receptor antagonists | None | 6 | 0.0 |
| 6 | Integrin receptor antagonists | Censored | 10 | 0.1 |
| 6 | IL-12/23 inhibitors | Conventional treatment | 3 | 0.0 |
| 6 | IL-12/23 inhibitors | TNF-alpha inhibitors | 3 | 0.0 |
| 6 | IL-12/23 inhibitors | Integrin receptor antagonists | 4 | 0.0 |
| 6 | IL-12/23 inhibitors | IL-12/23 inhibitors | 222 | 1.7 |
| 6 | IL-12/23 inhibitors | None | 20 | 0.2 |
| 6 | IL-12/23 inhibitors | Censored | 41 | 0.3 |
| 6 | None | Conventional treatment | 634 | 4.8 |
| 6 | None | TNF-alpha inhibitors | 72 | 0.5 |
| 6 | None | Integrin receptor antagonists | 4 | 0.0 |
| 6 | None | IL-12/23 inhibitors | 10 | 0.1 |
| 6 | None | None | 2494 | 18.8 |
| 6 | None | Censored | 439 | 3.3 |
| 6 | Censored | Censored | 2434 | 18.4 |

**Abbreviations:** IBD, inflammatory bowel disease; CD, Crohn’s disease; TNF, tumor necrosis factor; IL, interleukin; JAK, Janus kinase

^a^1: 0 to 6 months; 2: 6 to 12 months; 3: 12 to 18 months; 4: 24 to 30 months; 5: 30 to 36 months

^b^denominator: total number of treatment patterns in a specific time period; numerator: number of each pattern in a specific time period
